# Appropriateness and readability of Google Bard and ChatGPT-3.5 generated responses for surgical treatment of glaucoma

**DOI:** 10.22336/rjo.2024.45

**Published:** 2024

**Authors:** Parul Ichhpujani, Uday Pratap Singh Parmar, Suresh Kumar

**Affiliations:** Department of Ophthalmology, Government Medical College and Hospital, Chandigarh, India

**Keywords:** ChatGPT, glaucoma, Google Bard, Large Language Model, AI = Artificial Intelligence, LLM = Large Language Model, GPT-3 = Generative Pre-trained Transformer 3, SMOG = Simple Measure of Gobbledygook, IOP = intraocular pressure, GDD = Glaucoma Drainage Devices, AMA = American Medical Association, LaMBDA = Language Model for Dialogue Applications

## Abstract

**Aim:**

To evaluate the appropriateness and readability of the medical knowledge provided by ChatGPT-3.5 and Google Bard, artificial-intelligence-powered conversational search engines, regarding surgical treatment for glaucoma.

**Methods:**

In this retrospective, cross-sectional study, 25 common questions related to the surgical management of glaucoma were asked on ChatGPT-3.5 and Google Bard. Glaucoma specialists graded the responses’ appropriateness, and different scores assessed readability.

**Results:**

Appropriate answers to the posed questions were obtained in 68% of the responses with Google Bard and 96% with ChatGPT-3.5. On average, the responses generated by Google Bard had a significantly lower proportion of sentences, having more than 30 and 20 syllables (23% and 52% respectively) compared to ChatGPT-3.5 (66% and 82% respectively), as noted by readability. Google Bard had significantly (p<0.0001) lower readability grade scores and significantly higher “Flesch Reading ease score”, implying greater ease of readability amongst the answers generated by Google Bard.

**Discussion:**

Many patients and their families turn to LLM chatbots for information, necessitating clear and accurate content. Assessments of online glaucoma information have shown variability in quality and readability, with institutional websites generally performing better than private ones. We found that ChatGPT-3.5, while precise, has lower readability than Google Bard, which is more accessible but less precise. For example, the Flesch Reading Ease Score was 57.6 for Google Bard and 22.6 for ChatGPT, indicating Google Bard’s content is easier to read. Moreover, the Gunning Fog Index scores suggested that Google Bard’s text is more suitable for a broader audience. ChatGPT’s knowledge is limited to data up to 2021, whereas Google Bard, trained with real-time data, offers more current information. Further research is needed to evaluate these tools across various medical topics.

**Conclusion:**

The answers generated by ChatGPT-3.5™ AI are more accurate than the ones given by Google Bard. However, comprehension of ChatGPT-3.5™ answers may be difficult for the public with glaucoma. This study emphasized the importance of verifying the accuracy and clarity of online information that glaucoma patients rely on to make informed decisions about their ocular health. This is an exciting new area for patient education and health literacy.

## Introduction

The Internet of Medical Things, Telemetry, and Healthcare apps are virtually affecting every area of healthcare. With the birth of Dialogue-based Artificial Intelligence (AI) chatbots, which comprehend natural language and offer conversational-style replies, patients are now looking for relevant health-related medical information via these platforms.

The pioneer, OpenAI (San Francisco, USA), released ChatGPT, a generic Large Language Model (LLM) based on the Generative Pre-trained Transformer 3 (GPT-3) series, in November 2022 [1]. ChatGPT-3.5 and ChatGPT-4 are the latest versions available. Many other applications that recently introduced LLMs include Google’s Bard, Meta’s LLaMa 2, Bing Chat, Anthropic’s Claude 2.0, Stanford’s Alpaca, and Vicuna 13B [2]. These datasets are trained on an extensive text collection featuring over 400 billion words from the internet, including articles, books, and websites.

ChatGPT and Bard offer similar services, where users input a query and receive responses that mimic human conversation.

The impact of ChatGPT has been huge for the public and the research community. For many patients and their caregivers, these AI platforms act as a “*virtual health coach*” [3,4]. Therefore, these LLM chatbots must provide clear and comprehensive answers.

The medical field poses a significant challenge for LLMs because it demands advanced clinical reasoning, typically requiring years of training and experience.

Glaucoma is a chronic, progressive disease that results in irreversible loss of vision and visual field. Therefore, glaucoma patients and their care providers are anxious about losing sight [5]. Glaucoma is treated with topical or oral antiglaucoma medications, lasers, or surgery (or a combination of treatments) to reduce intraocular pressure in the eye and prevent permanent vision loss. Few studies have evaluated the performance of LLMs in ophthalmology, particularly in the glaucoma-related question-and-answer tasks area [6]. Therefore, the current study was planned to investigate the appropriateness as well as readability of the answers given by the two free and most used LLMs, ChatGPT and Google Bard, regarding the next step after medical and laser therapy, vision, surgical treatment for glaucoma [7]. Literature is sparse about the role of LLMs in disseminating information about glaucoma.

## Methods

The retrospective, cross-sectional study was conducted in January 2024 using Google Bard and ChatGPT-3.5. Since the study was essentially a Non-Human Subjects Research, it was exempt from IRB review.

We created a list of common questions related to the surgical management of glaucoma. Twenty-five questions were submitted to both the Google Bard and ChatGPT-3.5 online interfaces, with each question being asked three times to account for the possibility of different answers from these AI chatbots. These questions were about the surgical procedures available for glaucoma management in children and adults, indications, risk factors for surgical failure, mode of anaesthesia, surgical complications, success rate, and post-operative medications and follow-up. Two independent glaucoma specialists (PI and SK) evaluated each set of recorded responses for the questions, categorizing them as “appropriate”, “incomplete”, or “inappropriate” based on their clinical expertise.

An “appropriate” response was defined as one that closely aligns with the actual recommendations the reviewers would give their patients. An “inappropriate” response was either incorrect or deviated from the reviewer’s clinical recommendations. An “incomplete” response was considered relevant but lacking enough information. If the grading for all responses to a certain question was uniform, that grading was used as the final assessment. However, if the grades varied among consecutive responses, the final grading was marked as “inconsistent”.

To assess the readability of each response for a layperson, we input the answers into the online readability tool, Readable (https://app.readable.com/text/) [8]. The readability of each answer was assessed using seven different indices: the Flesch-Kincaid Grade Level, Flesch Reading Ease Score, Gunning Fog Index, Coleman-Liau Index, Automated Readability Index, Forecast Grade Level, and the Simple Measure of Gobbledygook (SMOG) Index [9,10].

The *Flesch-Kinkaid scoring systems* use sentence length and word length as parameters to assess the readability of the response. The Flesch Kincaid Grade level score refers to the United States grade level of education required to understand the body of text, and the Flesch Kincaid Reading Ease score is a score between 1 and 100 and a higher score is directly proportional to the ease of reading that text [11].

The *Gunning Fog Index* considers the number of complex words and the number of words in a sentence to generate a grade level between 0 and 20 [12].

The *Coleman Liau index* uses sentences and letters as variables to evaluate the reading level of a text [13]. It is used in addition to the other scores to assess particularly medical and law documents [14].

The *Automated readability index* gauges the understandability of a text by relying on the number of characters per word factor, instead of using syllables per word like the other indices [15].

The *Forecast grade level* focuses on functional literature and rather than relying on complete sentences, it analyses the number of single-syllable words in a sample to grade its readability [16].

The *SMOG score* uses the frequency of polysyllabic words as a tool to assess the readability of text. It has been shown very useful in the healthcare sector, to evaluate patient comprehension of healthcare-related terms [17,18].

The Coleman Liau, automated readability index, forecast grade level, and SMOG yield a score between 1 and 20 that is inversely proportional to the ease of readability of the text. The lower the score, the easier it is to read and comprehend the body of text.

### Statistical Analysis

The statistical analysis was conducted using R Studio (R Foundation for Statistical Computing, Vienna, Austria). The mean readability scores generated by Google Bard and ChatGPT-3.5 were compared using the student t-test. A statistical significance threshold of P < 0.05 was applied.

## Results

*1*. ***Appropriateness of the chatbot responses***

**Table 1** enlists the 25 questions the two reviewers posed to Google Bard and ChatGPT-3.5 and their gradings for the generated appropriateness of each answer. We used appropriate, inappropriate, and incomplete if all three responses to the same question had the same grading and inconsistent if there was any variation in the response grading when a question was asked three times.

**Table 1:** Appropriateness of the responses from Google Bard and ChatGPT-3.5 about the surgical management of glaucoma

Questions about the surgical management of glaucoma	Reviewer’s grading for Google Bard answer	Reviewer’s grading forChatGPT-3.5 answer
What are the surgical procedures for glaucoma in adults?	Appropriate	Appropriate
What are the surgical procedures for glaucoma in children?	Inappropriate	Appropriate
When to consider Trabeculectomy?	Appropriate	Appropriate
When to consider Glaucoma Drainage Devices?	Incomplete	Appropriate
When to consider Minimally Invasive Glaucoma Surgery?	Appropriate	Appropriate
When to consider Non-penetrating glaucoma surgeries?	Appropriate	Appropriate
Are there any risk factors for failure of glaucoma surgery?	Appropriate	Appropriate
Does cataract surgery lower intraocular pressure?	Inappropriate	Appropriate
What is the recovery time for a trabeculectomy, a Glaucoma Drainage Device, or tube-shunt surgery?	Appropriate	Appropriate
When to consider Bleb needling?	Appropriate	Inappropriate
Can glaucoma be cured after trabeculectomy or Glaucoma Drainage Devices?	Appropriate	Appropriate
Will a glaucoma surgery reverse all the damage that had been done?	Appropriate	Appropriate
Will a glaucoma surgery reverse all the damage that had been done?	Appropriate	Appropriate
Will I gain or lose vision after glaucoma surgery?	Appropriate	Appropriate
What is the mode of anesthesia for glaucoma surgery?	Appropriate	Appropriate
What are the common complications after Trabeculectomy?	Appropriate	Appropriate
What are the common complications after Glaucoma Drainage Devices?	Inappropriate	Appropriate
What are the common complications after Minimally Invasive Glaucoma Surgery?	Inappropriate	Appropriate
What are the common complications after non-penetrating glaucoma surgeries?	Inappropriate	Appropriate
What is the success rate of Trabeculectomy?	Appropriate	Appropriate
What is the success rate of Glaucoma Drainage Devices?	Appropriate	Appropriate
What is the success rate of Minimally Invasive Glaucoma Surgery?	Incomplete	Appropriate
What is the success rate of Non-penetrating glaucoma surgeries?	Incomplete	Appropriate
How long to take eye drops after a glaucoma surgery?	Appropriate	Appropriate

*2*. ***Google Bard:*** Appropriate answers to the posed questions were obtained in 68% (17/25) of the responses. Appropriate responses to questions regarding the surgical management of glaucoma included answers regarding the procedures available for adults when considering trabeculectomy, minimally invasive glaucoma surgery, and non-penetrating glaucoma surgery, the risk factors for surgery, recovery time after surgery, the common complications of trabeculectomy and in questions regarding the success rate and visual prognosis of these surgical procedures.

Responses were inappropriate for 20% (5/25) of the questions. These included responses to the mode of anaesthesia during glaucoma surgery, the common complications of non-penetrating glaucoma surgery, and when to consider bleb leak. For example, for the question regarding the surgical options for glaucoma in children, the statement that “*Children heal more slowly than adults, so it may take longer for them to recover from surgery.”* was incorrect.

To the question “Does cataract surgery lower intraocular pressure?”, *“Cataract surgery is a safe and effective procedure that can restore clear vision. It does not lower IOP directly, but it can indirectly lower IOP by improving vision. This is because improved vision allows people to see better and avoid activities that can raise IOP, such as straining to see.” was inappropriate*.

When queried “What are the common complications after Glaucoma Drainage Device surgery?”, the answer was “*Bleb failure: This is the most common complication of GDDs. It occurs when the bleb, which is the small, raised area of tissue that is created on the eye surface during surgery, does not work properly. This can lead to increased IOP and vision loss. Infection: Infection is another common complication of GDDs. It can occur at the incision site or in the bleb.”* was incorrect.

The same answer was repeated for *“What are the common complications after Minimally Invasive Glaucoma Surgery and Non-penetrating Glaucoma Surgery?”*, even though bleb is not formed in MIGS and NPGS.

Finally, responses were incomplete for 12% (3/25) of the questions. These included the ones that the Chatbot generated to the surgical procedures available for children, when to consider glaucoma drainage devices, common complications for glaucoma drainage devices and minimally invasive glaucoma surgery, how long to take eye drops after glaucoma surgery, and the questions about the success rate of minimally invasive glaucoma surgery and non-penetrating glaucoma surgery. For example, “When to Consider Glaucoma Drainage Devices”, would have been more informative if the types of glaucoma, where it was likely to work better than trabeculectomy, had been mentioned. The answer provided was generic and befitted both Glaucoma Drainage Devices and trabeculectomy. Concerning the success rate of all forms of surgery, common factors that can affect success are the severity of the glaucoma, the patient’s age and overall health, and the surgeon’s experience. Overall health has no direct bearing on surgical success, however, the type of glaucoma and ocular surface, which have a direct bearing, have not been mentioned.

***ChatGPT-3.5****:* The ChatGPT-3.5 artificial intelligence chatbot performed significantly better than Google Bard in generating appropriate responses for the questions posed.

Appropriate responses were obtained for 96% (24/25) of the questions. These included questions regarding the surgical procedures available when to consider these procedures, the risk factors of these procedures, recovery times after surgery, prognosis, common complications, and the success rate of these surgical procedures.

ChatGPT-3.5 included laser therapy also among surgical procedures. An inappropriate response was generated for the question “When to consider bleb needling?”. The answer that “*Bleb needling may be considered to repair the bleb leak and restore the bleb function*. ” was inappropriate.

***3. Readability of the responses:*** The average Flesch Kincaid grade level, Flesch reading score, Gunning fog score, Coleman Liau score, and SMOG score for the responses generated by Google Bard were 8.94±1.2, 57.91±7.1, 12.28±1.81, 9.55±1.02, 12.2±1.24 respectively. The average Flesch Kincaid grade level, Flesch reading score, Gunning fog score, Coleman Liau score, and SMOG score for the responses generated by ChatGPT-3.5 on the other hand were 15.4±1.80, 22.51±7.04, 18.59±2.26, 15.68±1.25, 17.05±1.63 respectively. The average scores generated by all the readability indices have been summarized in **Table 2**.

**Table 2:** Comparison of the readability indices between Google Bard and ChatGPT-3.5 recommendations about the surgical management of glaucoma

Scores	Google Bard		Chat GPT		P value
	Mean ±SD	Median	Mean ±SD	Median	
Flesch Kincaid Grade Level	8.94 ±1.2	8.8	15.4 ±1.80	15.8	<0.0001
Flesch Reading Ease Score	57.91±7.1	57.6	22.51 ±7.04	22.60	<0.0001
Gunning Fog Score	12.28 ±1.81	11.70	18.59 ±2.26	18.40	<0.0001
Coleman Liau Score	9.55 ±1.02	9.3	15.68 ±1.25	15.30	<0.0001
Simple Measure of Gobbledgook Index (SMOG)	12.2 ±1.24	12	17.05 ±1.63	17.10	<0.0001
Automated Readability Index	8.08 ±1.23	7.6	15.48 ±2.28	15.80	<0.0001
Forecast Grade Level	10.21 ±0.44	10.10	12.57 ±0.47	12.60	<0.0001

Google Bard had significantly (p<0.0001) lower readability grade scores and a quite higher “Flesch Reading ease score”, implying greater ease of readability amongst the answers generated by Google Bard. This could be attributed to the fact that on average the responses generated by Google Bard had a significantly lower proportion of sentences having more than 30 and 20 syllables (23% and 52% respectively) as compared to ChatGPT-3.5 (66% and 82% respectively) as noted by readability. The average number of characters per word and words per sentence for the answers given by Google Bard were 4.4 and 15.5 respectively, compared to the ChatGPT-3.5 responses, which had 5.9 and 24 respectively.

## Discussion

Globally, Glaucoma is the leading cause of irreversible vision loss. Public awareness about the available surgical options for glaucoma is not widespread, therefore, patients and care providers resort to the Internet to address their concerns [19]. Since the LLM Chatbots have taken the internet by storm, many glaucoma patients and/or their families have turned to these chatbots for answers. Therefore, correct information must be available in a lucid fashion. If the readability of the content is good, it can provide more fine-tuned guidance to the patients about what to expect from the disease and its treatment. It is imperative to gauge the generated responses’ precision, dependability, and readability [6,20].

In February 2024, Bard and Duet AI were consolidated under the Gemini brand, however, since the data was collected in January when it was still “Bard”, hence the use of this name in our manuscript.

Assessments of the quality of glaucoma-related information, available online, have been limited to websites and blogs evaluation. Jia et al. assessed fifteen of the most popular websites on the internet and found that online information about glaucoma varies in content, quality, accuracy, and readability [21]. Shah et al. reported that most websites evaluated had glaucoma-related information of low quality, reliability, and readability [22]. The authors found that institutional websites were significantly better than private websites. Khan et al. found that information available online for laser peripheral iridotomy and trabeculectomy is of variable and poor quality, putting the patient at risk of receiving incorrect information [23]. Cohen et al. conducted a Google search using 10 terms related to glaucoma, examined 200 websites, and discovered that only 11% were written at or below the recommended 6th-grade reading level. However, unlike our study, they did not assess health information from the currently trending LLMs [24].

The American Medical Association (AMA) advises that online patient education materials should be written at or below a 6th-grade reading level [25]. Readability refers to how easily a reader can comprehend a written text. Even though there are no similar comparisons for medical queries, experts believe that in general, ChatGPT-3.5 is more precise in responses, while Google Bard scores better in terms of readability. In our study also, readability was better with Google Bard as the Median Flesch Reading Ease Score for Google Bard was 57.6 and for Chat GPT it was 22.6 (A score of 0 to 30 indicates that the text is very troublesome to read and best understood by university graduates; a score of 30 to 50 suggests that the content is challenging and suited for college graduates; and a score of 50 to 60 means that the text is somewhat difficult to read.). Even the Flesch Kincaid Grade Level scores were average for Google Bard but fell in the skilled reading level range for Chat GPT. Easy readability may be attributed to **the data sources used to train the platforms’ LLMs**.

The Gunning Fog Index should generally be below 12 for texts intended for a broad audience. The index should be below 8 for texts that require nearly universal comprehension. The median Gunning Fog Index for Google Bard was 11.70 and for Chat GPT was 18.40. The median Coleman Liau index score for Google Bard was 9.3 (for people with school level from 8th grade to 11th grade) and for Chat GPT was 15.3 (for people with school level from 11th grade to college).

ChatGPT utilizes the Generative Pre-trained Transformer 4 (GPT-4), whereas Bard employs the Language Model for Dialogue Applications (LaMBDA). Bard was trained specifically to increase its dialogue and coding powers and has access to the internet in real-time, so it has up-to-date information. ChatGPT’s knowledge is based on data up to 2021, so it has limited information on more recent research [26,27].

The main limitation of our study was that it involved a small number of questions focused solely on glaucoma. Consequently, we could not determine if ChatGPT-3.5 would perform differently with other ophthalmic topics. More studies are needed to assess its recommendations by experts in other ophthalmic subspecialties and other medical specialties [28]. Furthermore, we employed subjective grading to evaluate the appropriateness of the responses, which means our findings might not apply to other chatbots and similar technologies.

## Conclusion

Further research is necessary to assess the clarity and acceptability of LLM-generated answers from the patient’s perspective and to identify the most ethical and effective ways to use LLMs to enhance health literacy.
